# Synthetic Fabrication of Nanoscale MoS_2_-Based Transition Metal Sulfides

**DOI:** 10.3390/ma3010401

**Published:** 2010-01-12

**Authors:** Shutao Wang, Changhua An, Jikang Yuan

**Affiliations:** 1 College of Chemistry and Chemical Engineering, China University of Petroleum, Dongying, Shandong 257061, China; E-Mail: shtwang@upc.edu.cn (S.W.); 2 College of Chemical Engineering and Materials Science, Zhejiang University of Technology, Hangzhou, Zhejiang 310014, China; E-Mail: jikangy@hotmail.com (J.Y.); 3 Department of Applied Physics, The Hongkong Polytechnic University, Hung Hom, Hongkong

**Keywords:** transition metal sulfide, molybdenum sulfide, fabrication, surfactant, polymer, support, promotion, doping, intercalation

## Abstract

Transition metal sulfides are scientifically and technologically important materials. This review summarizes recent progress on the synthetic fabrication of transition metal sulfides nanocrystals with controlled shape, size, and surface functionality. Special attention is paid to the case of MoS_2_ nanoparticles, where organic (surfactant, polymer), inorganic (support, promoter, doping) compounds and intercalation chemistry are applied.

## Contents

**1.**  **Introduction**
**2.**  **General Synthesis of Transition Metal Sulfides Nanoparticles**
  *2.1*. Structure
  *2.2*. *General* *Synthetic Strategy*
**3.**  **Synthetic Fabrication of Molybdenum Sulfide Nanoparticles with Organic Compounds**
  *3.1*. *Synthetic Fabrication of Molybdenum Sulfide with Surfactants*
    *3.1.1*. Soft Templates
    *3.1.2*. Structure Directors
    *3.1.3*. Structure Stabilizers
  *3.2*. *Synthetic* *Fabrication of Molybdenum Sulfide with Polymers*
**4.**  **Synthetic Fabrication of Molybdenum Sulfide Nanoparticles with Inorganic Compounds**
  *4.1*. *Synthetic* *Fabrication of Molybdenum Sulfide with Support*
  *4.2*. *Synthetic* *Fabrication of Molybdenum Sulfide with Promoter*
  *4.3*. *Synthetic* *Fabrication of Molybdenum Sulfide with Doping*
**5.**  **Synthetic Fabrication of Molybdenum Sulfide with Intercalation Chemistry**
**6.**  **Concluding Remarks**

## 1. Introduction

Transition metal sulfides, especially molybdenum sulfide (MoS_2_), are scientifically and technologically important materials. There has been increasing interest in the synthesis of these sulfides because of their potential applications is areas such as electrochemistry [1,2], lubrication [3,4,5,6,7], catalysis [8,9,10,11], and as host materials for intercalation chemistry [12,13,14,15]. The physical and chemical properties of bulk sulfides have been investigated extensively. In recent years, nanometer-sized sulfides, which are promising building blocks for engineering and for tailoring nanoscale structures, have been prepared using a variety of methods [16], but the development of novel or general strategies to fabricate the nanoscopic materials with specific size, morphology, and property remains a challenge for materials chemistry.

With high degree of synthetic control, nanocrystals will offer a highly tailored structure from the micro- and nanoscale to the molecular level [16], and have a profound impact for applications such as biomedical diagnostics and medicine or surface catalysis. Currently, the controlled synthesis or fabrication of structurally anisotropic MoS_2_ is of primary importance in heterogeneous catalysis, since the size and surface property of the particles are of the main parameters to obtain highly active and selective catalysts [17]. Experiments have shown that there are a wide variety of compounds that can facilitate shape control [16,18,19], including surfactants, polymers, small molecules (such as adsorbed gas), and even atomic species (such as metal ions). Numerous papers on this subject have been published. Recent developments in the shape control of colloidal metal and metal oxide nanocrystals have been reviewed by Yang and Jun *et al*., respectively [16,20]. However, the exact mechanism(s) for controlled synthesis are often not well understood or characterized and precise tuning of nucleation and growth steps is needed to achieve crystallographic control. For example, Wang *et al*. reported a unified approach to produce a large variety of nanocrystals with different chemistries and properties and with low dispersity through liquid–solid–solution (LSS) phase transfer and separation strategy [21]. Puntes *et al*. have successfully controlled the size and shape of anisotropic cadmium selenide nanorods, magnetic cobalt nanorods as well as spherically shaped nanocrystals, separately [22].

The discoveries of carbon and inorganic fullerenes (IF) of layered metal dichalcogenides MX_2_ (M = Mo, W and X = S, Se) have stimulated multi- and interdisciplinary research activities for a wide spectra of possible applications [7,23,24,25,26,27,28]. Weak van der Waals forces between the molecular layers and strong intralayer covalent bonds are necessary for the formation of these unusual nanoparticles [29]. The state of the field has been reviewed by Tenne [4]. MoS_2_ and WS_2_ fullerene nanoparticles are valuable materials for many applications where dynamic pressures and high temperatures are involved [25]. They showed excellent antishock or shock-absorbing property under very high shock wave pressures of 25 GPa, accompanied with high concurrent temperature up to 1,000 °C. At the same time, these fullerenes may suffer destruction under high localized pressure and temperature *via* two possible mechanisms, *i.e.,* direct stress-induced breakage failure and diffusion-controlled oxidation, separately or combined. A combination of experiments and density functional tight binding calculations with molecular dynamics annealing are used to elucidate the structures and electronic properties of nanooctahedral MoS_2_ fullerenes [30], which are believed to be the smallest stable genuine inorganic fullerenes.

Progress has also been made in the exciting direction of the expansion from single component materials to hybrid materials [31,32,33,34], which would thus integrate or combine synergistically different functions in a controlled fashion. Sometimes, even entirely novel structures and properties could be provided *via* the coupling between the individual components of the hybrids. Thus multifunctional hybrid nanoparticles that combine magnetic, plasmonic, and semiconducting properties and those are tunable in size and morphology will be realized. Recent work on the polymer-assisted fabrication of well-defined inorganic and organic hybrid nanoparticles with controlled shape, size, and functional properties has been reviewed by Rozenberg and Tenne [31]. Castelvetro *et al*. have reviewed the basic definitions and the main synthetic routes available for the nanostructured organic-inorganic hybrid core–shell nanoparticles [32]. Shi *et al*. have described a general strategy for engineering binary and ternary hybrid nanoparticles (combinations of Au, Fe_3_O_4_, and PbS or PbSe) based on the spontaneous epitaxial nucleation and growth of a second and third component onto seed nanoparticles in high-temperature organic solutions [33].

The combination of different materials could produce improved mechanical, chemical, electrical, magnetic and optical properties. These properties depend upon the distribution and the composition of basic phase in the composite materials [35]. However, it is still a challenge to precisely control the composition, size and morphology of the object products with good reproducibility, since both nucleation and growth of the nanocrystals only happen in a homogeneous system, especially when the reaction rate was too fast [36]. With improvements in electron microscopy techniques and instrumentation, new experimental insights would be gained regarding the kinetics of nanocrystal growth [16]. Despite the very different chemistry involved, the reported fabrication process can be classified into two categories: by grafting desired groups to the surface of nanomaterial after synthesis (post-functionalization) or by the modification of nanomaterial *in situ* in certain solvents (aqueous or non-aqueous) in the presence of dissolved electrolytes, surfactants, or polymers (*in situ* functionalization) [18].

This article presents an overall picture of the fabrication of transition metal sulfides, with a particular focus on the solution-based surfactant assisted synthesis of MoS_2_ nanoparticles. This paper cannot be comprehensive because of the large amount of literature. Rather, we would like to focus on some remarkable features of the current subject. The fabrication strategy is divided into several groups, sometimes arbitrarily and subjectively. For instance, some surface active polymers are classified as polymers, not surfactants. Intercalation of the supported sulfides with organic molecules is considered as a method of intercalation fabrication. Supported sulfides promoted with nickel or cobalt is viewed as a promotion fabrication. This paper is made up of six sections. Section 1 and Section 2 provide the overall background for the subject and the general strategy for the synthetic control of transition metal sulfides. Parts 3 and 4 present an overview of different fabrication strategies, which are realized with organic (surfactant and polymer) or inorganic chemicals (support, promoter, doping), respectively. Part 5 describes the fabrication method with intercalation chemistry. Finally, the outlook and challenges are discussed.

## 2. General Synthesis of Transition Metal Sulfides

### 2.1. Structure

In recent years, a great deal of efforts have been devoted to the effective synthesis and fabrication of transition metal chalcogenides, in particular, the study of nano-hydrid materials with novel structures and functions.

**Figure 1:**
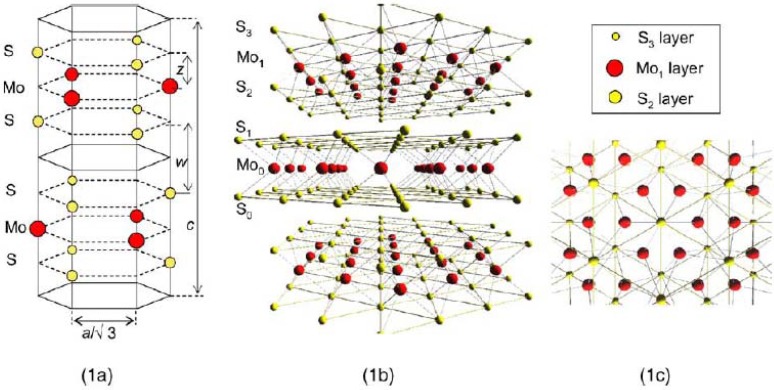
Crystal structure of MoS_2_ (1a), side view (1b) and simple projection of one sandwich S_2_–Mo_1_–S_3_ layer seen from Mo atoms lying in Mo_0_ layer for multi-domain surface (1c). Reproduced from reference [39] with permission from Springer.

Because of the unfilled *d*-shell of transition metals, compounds formed with transition metals exhibit novel and advantageous catalytic, optical and electronic properties distinct from those formed with non-transition metals. Furthermore, transition metal chalcogenides exhibit a wide variety of nanostructured allotropes with varying dimensionality and competing internal structure [37,38], such as nanorods, nanostripes, nanotubes, fullerene-like particles and fullerenes from the mesoscopic to nanoscopic scale. Enyashin *et al*. reviewed the rich polymorphism, structure, and the derived analysis of the reactivity and application potential encountered on different length scales [39]. By using two-dimensional display-type spherical mirror analyzer and circularly polarized lights [37], the stereoscopic photographs of the hexagonal arrangements of atoms Mo and S in MoS_2_ single crystals had been obtained successfully. The unique sandwich and the long interlayer structures had been confirmed, as illustrated in Figure 1.

To develop a fundamental understanding of the active sites of MoS_2_ catalysts in the hydrotreatment process, an *ab initio* investigation of the structural and electronic properties of a real size stoichiometric Mo_27_S_54_ cluster has been performed [40]. The electronic properties of the atoms at various sites (*i.e.,* corner, edge, outer, and inner positions) have been distinguished clearly by means of charge distribution and molecular orbital calculations. The corner S atoms had the most negative charge, and the corner Mo atoms possessed the most positive charge, suggesting that the atoms on the corners of a real MoS_2_ catalyst showed exceptionally reactive features for the hydrotreatment reactions.

### 2.2. General Synthetic Strategy

Generally, transition metal sulfides are prepared by direct combination of the elements or gas solid reactions between unsupported/supported oxides and a reducing H_2_S or H_2_S + H_2_ atmosphere at elevated temperatures [41]. These methods involve high temperatures, multiple procedures, or complex apparatus. Morphology control is also difficult in such preparations because of the topotactic relationship between the precursors and the produced sulfides [41]. Moreover, the surface of the sulfide is always partially hydrolyzed and oxidized [42]. Therefore, a dramatic decrease of the specific surface area of the powders could happen. Recent studies by French scientists have reviewed the preparation methods, activation procedures and design of active phases and supports for molybdenum or tungsten sulfide based hydrotreating catalysts [10]. A great variety of synthetic methods of the sulfides at relative low temperature are available, such as electrochemical deposition [43,44,45], laser ablation [30], microwave [27,46], molten salt [47,48], chemical vapor deposition (CVD)—metal-organic chemical vapor deposition (MOCVD) [49], and sonochemical preparation [50,51,52]. It was noted that the sonochemically prepared MoS_2_ exhibited improved catalytic performance, despite a much lower surface area compared to the conventional one [52], which was due to a particular morphology of the MoS_2_ slabs characterized by a high content of edges and defects induced by sonolysis.

In recent years, extensive studies on the solution phase synthetic routes to modify the morphology and thus property of the nanocrystals have been carried out. The solution process generally involves several consecutive stages: Nucleation from initially homogeneous solution, growth of the pre-formed nuclei, isolation of particles from the reaction mixture after they reached the desired size [53]. This route is also called the soft chemistry (chimie douce) route, which is particularly interesting since it does not require extreme conditions of pressure and temperature. Recently, Ma *et al*. synthesized MoS_2_ nanostructures with different morphologies *via* a facile ionic liquid-assisted hydrothermal route using 1-butyl-3-methylimidazolium tetrafluoroborate or 1-butyl-3-methylimidazolium hexafluoro- phosphate as an additive [54]. It was found that the amount and species of the ionic liquid played a crucial role on the sizes and morphologies of the MoS_2_ nanostructures, which were assembled by nanosheets and had potential applications in electrode materials and super solid lubricants. Precipitation of the corresponding sulfides can be easily achieved through reactions of chemically homogeneous precursors using H_2_S, Na_2_S, CS_2_, CH_3_CSNH_2_, CSN_2_H_4_, and KSCN, *etc*. as sulfurization reagents at much lower temperatures. Furthermore, solvothermal decomposition results in nanostructured material distinct from that obtained by decomposition of the precursor in sealed quartz tubes at the same temperature [55]. Pan *et al*. found that thiourea with a slow decomposition rate was a more preferred sulfur source than highly reactive Na_2_S, since the control of the nanocrystal size by reaction time must be realized with a slowed down reaction rate [36]. The reduction of transition metal ions by hydroxylamine hydrochloride (NH_2_OH·HCl) or hydroxylamine sulphate (NH_2_OH·H_2_SO_4_) to produce metal sulfides is also ubiquitous [43,44]. MoS_2_ derived from the aqueous preparations presents advantageous textural properties with respect to the samples prepared by thermal decomposition of ammonium tetrathiomolydbate [ATTM, (NH_4_)_2_MoS_4_], or other thiomolybdate precursors. The molecular precursors of thiosalts and thiosalt complexes are interesting alternatives for the preparation of sulfide materials with controlled morphology, because their decomposition has been reported to undergo a topotactic transformation [17,56]. Decomposition of ATTM, partially oxygen-substituted MoOS_3_^2^^－^ MoO_2_S_2_^2^^－^ and MoO_3_S^2^^－^ ions, and other sulfur containing coordination precursors would result in high-activity and high-surface-area MoS_2_ under vacuum, inert atmosphere or in certain solutions [26,42,57,58,59,60]. Thermal decomposition of ammonium salts of MoS_4_^2−^ and Mo_3_S_13_^2−^ within the confined voids of a porous aluminum oxide template would result in nearly monodisperse microscopic nanofibers and nanotubules of MoS_2_ [61]. Afanasiev and coworkers prepared high surface area MoS_2_ single layers using reactions of aqueous ATTM with N_2_H_4_ or NH_2_OH·H_2_SO_4_ [58,62]. Strong impact of the decomposition conditions such as heating rate, final temperature, gas nature, its pressure and its flow rate was observed [26]. It is interesting that the addition of water is also effective for generation from ATTM in the presence of *n*-tridecane solvent of highly active Mo sulfide catalysts which are very beneficial for the cleavage of C–C bond and hydrogenation (HYD) of aromatic moieties [59]. Unfortunately, the preparation of thiomolybdate precursors can be laborious and industrially unfeasible [60], since toxic and expensive ammonium sulfide or even worse, H_2_S bubbling are needed.

**Figure 2:**
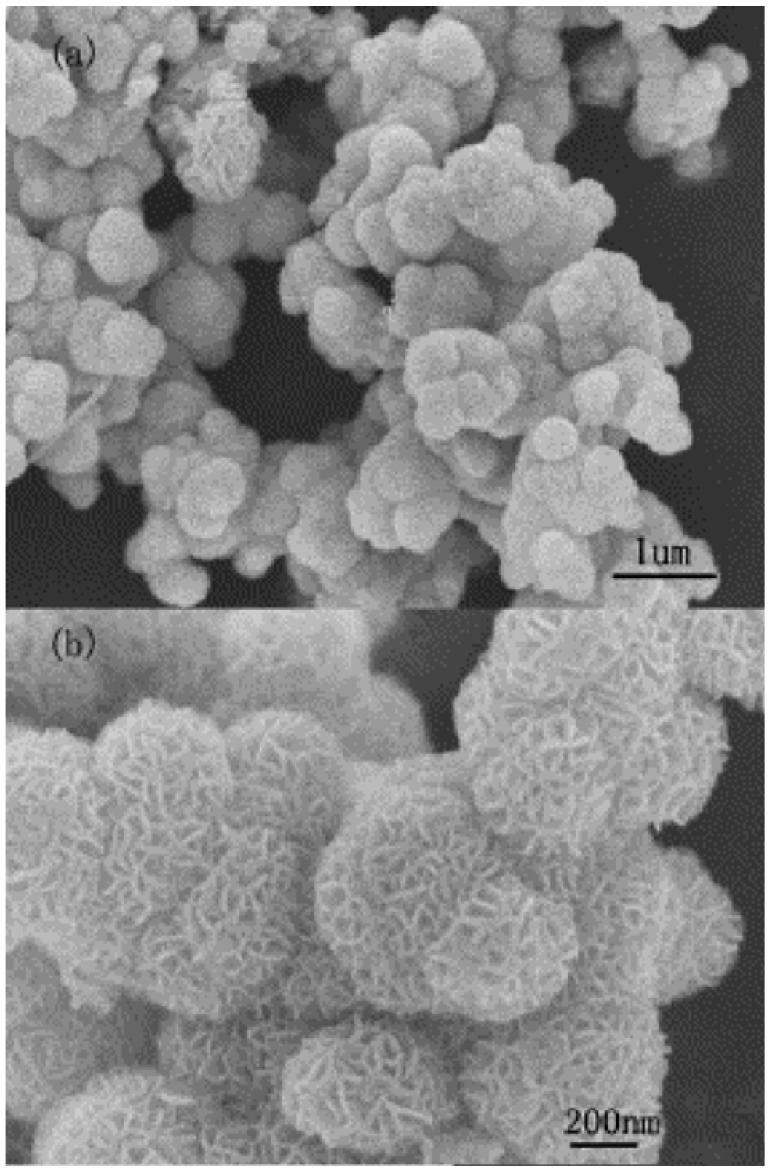
Morphology of the submicrometer MoS_2_ spheres. (a, b) FESEM images. Reproduced from reference [66] with permission from Elsevier.

Lately, the synthetic aspects of the MoS_2_-based catalytic materials have been reviewed [26], emphasizing the relationship between the preparation techniques (molecular precursor decomposition, hydrothermal, soft chemistry aqueous, surfactant-aided, intercalation-exfoliation, and solid-gas reactions) and the properties of the materials obtained. Although much progress has been made, it seems that synthesis of highly reactive MoS_2_ with large surface area, thermal stability and mechanical strength remains a challenging task, and amorphous and unstable powders are usually obtained [43,56,63]. Aqueous hydrolysis or acidification of MoS_4_^2^^－^ species lead to molybdenum trisulfide MoS_3_, but MoS_3_ can only be prepared in the amorphous form in the low pH range, which is easy to confuse with poorly crystallized substoichiometric sulfides [26,43], making it difficult to elucidate the exact structure using conventional X-ray diffraction analysis. The structure of MoS_3_ obtained by various solution methods might be different, while electrochemically and thermally produced MoS_3_ are structurally identical. However, additional sulfur is present in the electrodeposited material [43], suggesting that different chemical processes are involved in each procedure and the elemental sulfur remains entrapped in the MoS_2_ film during the electrochemical process. Tian *et al*. proposed a reduction and subsequent sulfidation mechanism for the hydrothermal synthesis of amorphous MoS_2_ nanospheres [64]. They found that phase pure MoS_2_ could only be obtained when the pH value is between 6.0 and 7.5. Rice *et al*. prepared polycrystalline MnS, FeS_2_, and amorphous sulfur-rich sulfides of Cr, Mo and W by the reaction of the transition-metal carbonyls with sulfur in 1,2-dichlorobenzene [65]. High quality binary sulfides of WS_2_, MoS_2_, and V_5_S_8_ have been prepared *via* a direct sulfurization route from the respective oxides *via* (hydro)solvothermal syntheses [66]. The produced MoS_2_ has a flower-like morphology with an average diameter of 400 nm, the surface of which composed of nanoflakes with a width of ca. 20 nm (Figure 2) [66].

**Figure 3:**
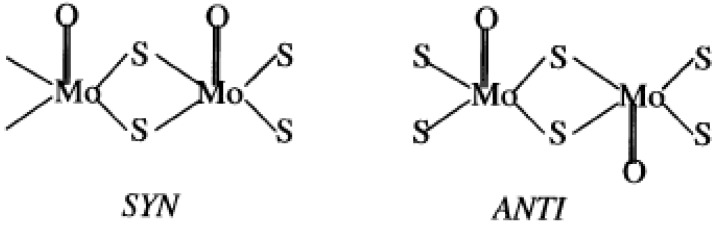
Possible configurations in the molybdenum oxysulfide species. Reproduced from reference [67] with permission from the American Chemical Society.

Binary molybdenum sulfides more rich in sulfur, MoS*_x_*, MoS_5_ or MoS_6_, might be available from the acidic hydrolysis of (NH_4_)_2_Mo_2_S_12_ (ammonium thiodimolybdate, ATDM) [56,57]. In reference [67], amorphous molybdenum oxysulfides with a variety of original morphologies, including entangled tubules, hollow spheres, and sponge-like fractal structures, were obtained in an acetone−water mixed solvent. The dominating type of morphology of the amorphous oxysulfides can be controlled by variation of the ionic strength of the reaction mixture with varying amounts of electrolytes, such as NH_4_SCN, KCl, (NH_4_)_2_SO_4_, or (NH_2_OH_2_)HSO_4_, without any external surfactant involved. Possible configurations in the molybdenum oxysulfide species were illustrated in Figure 3. A new type of amorphous sulfur microtubules was prepared from ATDM in acetone solution without any organic structure-directing agents [68]. It was supposed that the oligomeric species formed from Mo_2_S_12_^2-^ anions with different polarities behaved similarly in solution to organic surfactant molecules, played a key role in the formation of the microtubules. The sulfur molecules can be incorporated in the less polar domains of the condensation products of thiomolybdate oligomeric species, resembling the solubilization of nonpolar organic molecules in the hydrophobic domain of surfactant-formed structures.

In recent years, IF nanoparticles and nanotubes have attracted considerable interest. Details of the growth mechanism of IF-MoS_2_ by a gas-phase reaction from MoO_3_ powder had been reported by Zak *et al.* [29], where they found that the formation of the suboxide MoO_3-*x*_ nanoparticles as precursors for IF-MoS_2_ was ascribed to a partial reduction of (MoO_3_)_3–5_ molecular clusters with hydrogen. They also found that the parameters influence the formation of the nanoparticles were the temperature, flow rates of gases, concentration of gases (H_2_ and H_2_S), and reactor construction (nozzle’s size and shape position of the oxide precursor, tube’s diameter, *etc*.)

## 3. Synthetic Fabrication of Molybdenum Sulfide with Organic Compounds

Usually, the industrial catalytic system is triphasic with a solid catalyst, reducing gas, and a liquid phase of complex mixtures of hydrocarbons. The efficiency of traditional MoS_2_ based HYD catalysts is rather low because of the large particle size and poor solubility in oil phase. The tribological application of MoS_2_ as lubricating additives is also limited by its insolubility in the lubricating oils. As a promising substitute, nanoscale sulfide has been extensively investigated, but the nanoparticles would suffer from particle aggregation and surface oxidation because of the high surface energy. Generally, the nanospheres would grow into large particles *via* the mechanism of Ostwald ripening, whereby the smaller particles dissolve and are consumed by the large particles, leading to increased average nanocrystal size and decreased particle concentration [53]. To increase the dispersion of the sulfide nanoparticles, sonochemical and other high energy irradiation methods have been applied, involving specials apparatus, tedious procedure, or high temperature. Mdleleni *et al*. prepared high-surface-area MoS_2_ particles with an average diameter of 15 nm by irradiating a slurry of molybdenum hexacarbonyl and sulfur in 1,2,3,5-tetramethylbenzene (isodurene) with high-intensity ultrasound (20 kHz) under Ar atmosphere [51]. Their study indicated that the sonochemical MoS_2_ exists as a porous agglomeration of spherical clusters, which are themselves aggregates of smaller particles.

Moreover, it is difficult to synthesize oil-soluble nanocrystals with water-soluble precursors in aqueous phase or organic phase [36]. Recently, the study of solid surfaces with controllable wettability has emerged as an exciting topic in surface science [69,70]. Surface organometallic chemistry (SOMC) has been applied to prepare materials with desired compositions and structures [71]. The graft (attachment, capping, or coating) of reactive organic groups on the surface of the inorganic nanomaterial by strong covalent or ionic interactions is an effective route to tailor the size and solubility of the hybrid particles [17,64]. The inorganic cores would thus be endowed with unique properties, such as wettability, physicochemical stability, dispersivity, biocompatibility, and optical properties. Bakunin *et al*. reviewed the state of art in the field of synthesis of inorganic nanoparticles and their applications in tribology [72], with special attention paid to those of surface-capped and bare MoS_2_ nanoparticles. Yu *et al*. prepared a Langmuir-Blodgett (LB) film of MoS_2_ nanoparticles coated with dialkyldithiophosphate (DDP) through ion modification technique, though it was difficult to form LB films of MoS_2_ nanoparticles alone [3]. It has been found that the DDP modified MoS_2_ showed a considerably decreased friction coefficient and increased wear resistance than the LB films of various fatty acids in sliding against GCr15 bearing steel. The improved tribological behavior of DDP coated-MoS_2_ nanoparticles was attributed to the lubricity of MoS_2_ nanocores and the DDP modifier with strong hydrophobicity [3,6]. The additional functional groups of the capping molecules of the nanocrystals provides their water solubility, high processability, and surface charge desired.

The use of surface organics as dispersing and protecting agents will increase the stability against aggregation and dispersibility of the particles in the non-polar solvents or polymer matrix. Such surfaces may be useful for the attachment of bimolecular or desired functional groups. It is well documented that the catalytic activity of MoS_2_ is localized at the edges, where sulfur vacancies are formed, and not on the flat basal planes. However, it seems that the fabrication of MoS_2_ with organic chemicals would not bring any increase of the edges amount of the MoS_2_ slabs. Besides the surface modification, so, it is especially attractive to design surface lipophilic catalysts with dramatically improved catalytic activity. On the other hand, heat treatment of the organic-inorganic hybrid materials would lead to porous or reticulate sulfides, beneficial for the goal to gain highly active catalysts with high surface area. Future investigation of the nanocomposites with specific and tailored properties are necessary, especially for the case of surfactant or polymer mediated fabrication of phase pure sulfide nanocrystals.

### 3.1. Synthetic Fabrication of Molybdenum Sulfide with Surfactants

Recently, controlled synthesis of nanomaterials with the assistance of surfactants is becoming a rapidly developing field. The selection of the proper surfactant is a key factor. The interactions between the surfactants and nanomaterials should be kept moderate. If it is too strong, the catalysts would undergo deactivation, while if too weak, destabilization would arise. There are different viewpoints about the role of surfactant incorporation. Afanasiev and coworkers studied the effect of the surfactant on the morphology and stability of molybdenum sulfide and found that the addition of the cetyltrimetylammonium chloride to the reaction mixtures led to an increase of specific surface area up to 210 m^2^/g, disappearance of MoS_2_ layer stacking and improved thermal stability [58]. Sometimes, the introduction of surfactants will endow heterogeneous character to the reaction, and even bring about a completely different reaction mechanism. The reduction of [MoS_4_]^2−^ anions with aqueous N_2_H_4_ under mild conditions usually results in the easy formation of MoS_2_. This reaction pathway was completely changed by the presence of the organic CTA^+^ cations, leading to a layered mesophase containing [Mo_2_O_2_S_2_(S_2_)_2_]^2−^ anions [73].

Particles with controllable size and shape can be obtained by varying the experimental parameters. By dynamically coating the particles with a close-packed monolayer of coordinating ligand, the surfactant has the ability to selectively control the growth rates of different faces and thus the size and shape of the growing particles [22]. In addition, the surfactant layer will prevent agglomeration of the nanoparticles, allow monomers to add or subtract, passivate the nanocrystals against oxidation, and define the minimum interparticle distance. A thorough comparative study of the role of surfactants would be fruitful, which are proposed to be multifunctional and will be discussed in the following parts in detail.

#### 3.1.1. Soft Templates

The surfactant molecules possess a hydrophilic head group and a hydrophobic tail, and can be readily self assembled into spherical or rodlike micelles in water, when the concentration of the surfactant reaches a value slightly higher than the critical micelle concentration (CMC). Such micellar structures are consisted of nanometer size droplets with insoluble cores and soluble shells. They provide an interesting reaction template as nanoreactors for the synthesis of nanoparticles with controllable diameter [16,17,74]. The formation of metal-surfactant complex during the reaction process might kinetically hinder the metal reduction in or around the spatially confined hydrophilic volume, and thus favor the formation of nanoparticles with a narrow size distribution.

**Figure 4:**
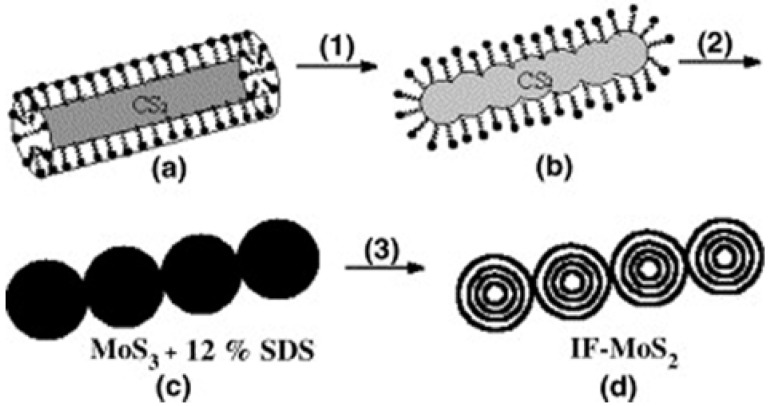
Schematic illustration for the formation of fullerene-like MoS_2_ nano-necklace: (1) SDS aggregations transform from rod-like to spherical micelles with the increase of temperature. (2) The necklace-shaped assembly of amorphous MoS_3_ nanospheres with 12 wt % SDS forms is realized by the aggregation transformation of surfactants. (3) The necklace-shaped assembly of MoS_2_ fullerene-like structures produces from the amorphous MoS_3_ nano-necklace, after removing the residual SDS by annealing treatment. Reproduced from reference [75] with permission from Elsevier.

Organic surfactants such as tetraalkylammonium species have been successfully applied as templates for the preparation of various mesoporous materials. Flores-Ortiz *et al*. obtained polycrystalline tubulenes of MoS_2_ by precipitating solutions of ATTM using two different tetraalkylammonium surfactants as templates, hexadecyltrimethylammonium bromide [CH_3_(CH_2_)_15_N(CH_3_)_3_Br] (CTAB) and tetrabutylammonium hydroxide [(CH_3_(CH_2_)_3_)_4_NOH; (But)_4_NOH) [56]. They found that the surfactant- templated hybrid phase would decompose at temperatures above 600 °C, yielding highly porous MoS_2_ particles with sponge-like morphology comprised of disordered pores between 0.1 and 3 μm in size. Well-aligned bundles of MoS_2_ tubulenes were obtained when tetrabutylammonium hydroxide was used as template [56]. This was explained by the weak interaction established between the surfactant and the thiosalt precursor, which allows the organization and orientation of the basal planes of the small S-Mo-S layers. The micelle-assisted of necklace-shaped assembly of inorganic fullerene-like molybdenum disulfide nanospheres has been reported (Figure 4) [75]. The aggregations of sodium laurylsulfonate (SDS) with a concentration ten times high as its CMC will offer microreactors for the reactions and the assemble of amorphous MoS_3_ nanospheres, which will then transform to the target fullerene-like MoS_2_ by annealing. Without adding the anionic surfactant SDS, the as-obtained products are some irregular nanoparticles without assembling.

**Figure 5:**
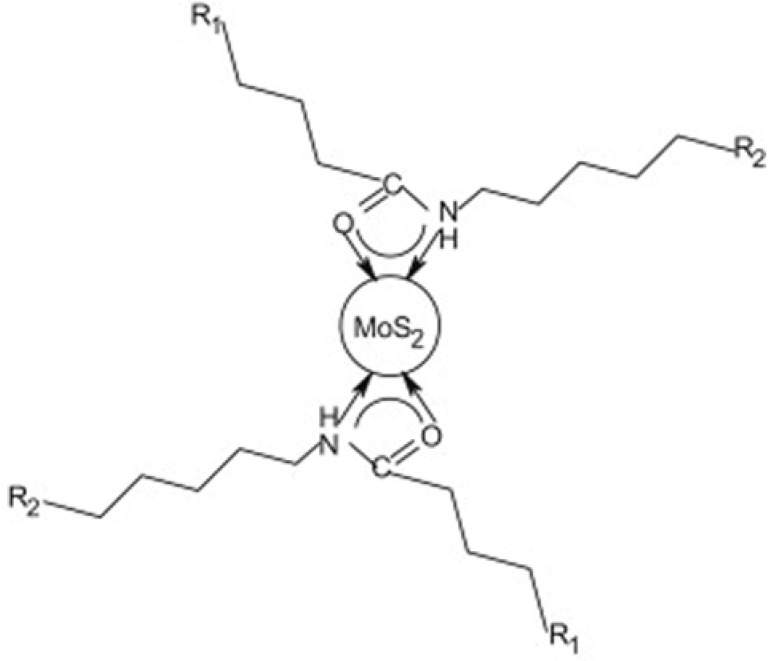
Structure model of the surface modified MoS_2_ nanoparticles. Reproduced from reference [76] with permission from Elsevier.

In addition to the concentration factor, the formation of the micelles also depends on the presence of other additives, such as cosurfactants. But in these mixed solutions, the role of surfactant adsorption is complicated due to the presence of stabilizing anions or other metal ions, which will modify the metal reduction even with low concentrations [16]. In reference [67], the authors showed that the morphology of the amorphous MoS*_x_* (and MoS_2_ further obtained from it) might be controlled by the change of the ionic strength of the reaction mixture. Recently, our group developed a generalized and green composite-surfactants-assisted-solvothermal process (CSSP) to produce highly homogeneous dispersive nanoparticles of metal sulfides (MoS_2_, CdS, MnS, Ag_2_S, PbS, FeS_2_, and NiS_2_) with various sizes and shapes [76]. These sulfides have a lipophilic surface and can form stable colloid solutions in organic solvent, such as heptane, dimethylformamide, and others. A possible in-situ reduction-sulfidation mechanism has been proposed to clarify the surface modification process. The surface of the MoS_2_ nanoparticles was suggested to be bonded to the surfactants sodium oleate (SOA) and hexadecylamine (HDA) through amide bonding (Figure 5).

**Figure 6:**
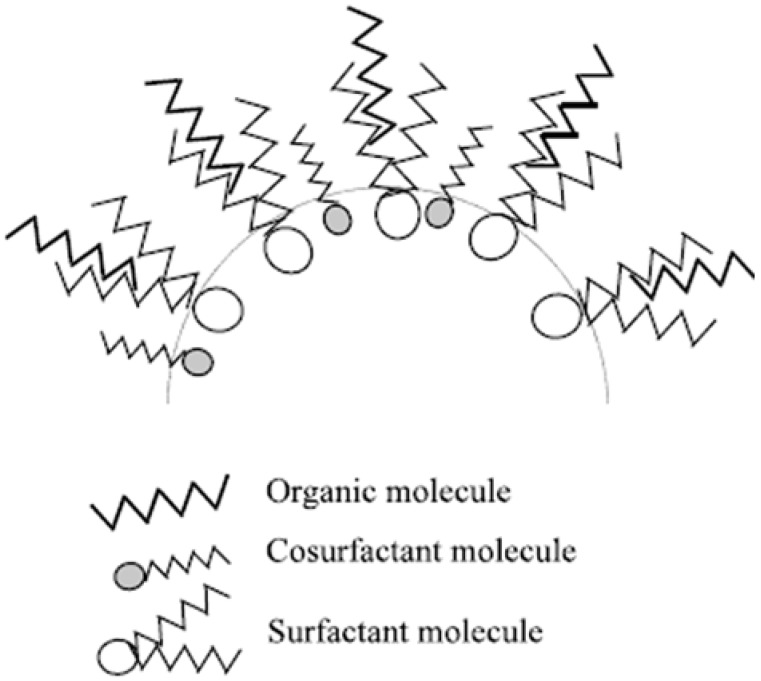
Schematic representation of an interface including surfactant and cosurfactant. Reproduced from reference [17] with permission from Elsevier.

Dodecanethiol-capped CdS nanoparticles were obtained in a Winsor II microemulsion of is (2-ethylhexyl)sulfosuccinate (AOT)/diethyl ether/H_2_O [74]. The Winsor type II system offers the possibility to synthesize nanoparticles of transition metal sulfides in large-scale using far higher salt concentrations than that of the conventional systems. The role of the anionic surfactant was to form the microemulsion and facilitate the extraction of oppositely charged ions from the aqueous to the organic reverse micellar phase. Marchand *et al*. presented a controlled synthesis of MoS*_x_* nanoparticles through acidification of ATTM contained in the microreactors made of water-in-oil microemulsions of AOT/water/*n*-heptane [17]. The reverse microemulsion phase was characterized by transmission electron microscopy (TEM) after freeze fracture and replication. The introduction of polyoxyethylene (5) nonylphenylether (NP-5) as a nonionic cosurfactant at a small concentration would result in a significant decrease of the microemulsion droplet size and thus the size of MoS*_x_* nanoparticles from the mean size of 8 nm to 4 nm. This decrease could be attributed to the higher fluidity of the microemulsion interface and the higher mean curvature of the interfaces of the micelles [17]. The modification of micellar interface by addition of a cosurfactant has been schematically represented by the following Figure 6 [17]. The study of various parameters such as the water-to-surfactant molar ratio and the ionic strength of the water cores have been conducted. It was found that AOT favored the formation of spherical water-in-oil droplets at low water content, but on increasing the water-to-surfactant molar ratio, interconnected cylinders would generate [17].

Recently, the application of organic surfactants or some inorganic electrolytes has been proven to be an interesting and effective strategy for inorganic syntheses [21,77]. Relatively few papers have dealt with VI B group sulfides, probably because of the lack of slow solution reactions which could be conveniently modified by surfactant introduction [26]. Moreover, the removal of the organic template usually involves an oxidation step either by air calcination or by reactions with some strongly oxidizing species. So, subsequent elimination of the template without destruction of the structure remains a difficult task.

#### 3.1.2. Structure Directors

Recently, one-dimensional nanomaterials have attracted extensive attentions because of their potential practical and theoretical applications. Lately, a number of studies to induce anisotropic growth or orientation of inorganic crystals with surfactants acting as structure directing agents have been reported. The general strategy to generate shape anisotropy during nanocrystal growth is through stabilizing a particular facet by employing molecular capping agents that selectively adsorbed to specific crystal planes [16]. Therefore, the targeted structure with preferential growth along certain orientation will be realized, since the crystal growth will be limited on the planes where binding is strong and promoted on the planes where binding is weak.

Tian *et al*. have successfully synthesized MoS_2_ nanorods through the reduction of ammonium molybdate ((NH_4_)_6_Mo_7_O_24_·4H_2_O) by sodium sulfide (Na_2_S·9H_2_O) and NH_2_OH·HCl in the presence of the surfactant sodium dodecyl benzene sulfonate (DBS) [78]. It has been found that the reduction product of Mo(VI) by NH_2_OH·HCl strongly depends on the reaction temperature and pH value of the solutions. The author suggested that the presence of DBS resulted in the epitaxial growth of the product during the formation of MoS_2_ nanorods. But in another work of their group, amorphous MoS_2_ nanospheres were resulted in almost the same reaction conditions [64], so further study and detailed discussion are necessary to clarify the crystallographic mechanism and the role of DBS surfactant.

The use of thiols as capping agents in the syntheses of II-VI semiconductor nanocrystals allowed the preparation of extremely small molecular-like clusters of exact composition [53]. Efforts are made to investigate the detailed structural characteristics of DDP-MoS_2_ obtained by an ion modification method [79], where the results show that the modified MoS_2_ nanoparticles are aggregates composed of 3~5 MoS_2_ nanocrystallites with a single layer of DDP molecules capped around the MoS_2_ cores. Zhou *et al*. obtained MoS_2_ micrometer spheres modified by the self-prepared surfactant, quaternary ammonium salt of 2-undecyl-1-dithioureidoethylimidazoline, with better dispersing capacity and tribological properties in liquid paraffin than commercial colloidal MoS_2_ [6]. The boundary lubrication mechanism is suggested based on the protective chemical adsorption film formed by the long chain alkyl and active elements (S and N) in the surface layer of the surfactant, and the tribochemical reaction film composed of the tribochemical reaction products of the additive [6].

#### 3.1.3. Structure Stabilizers

**Figure 7:**
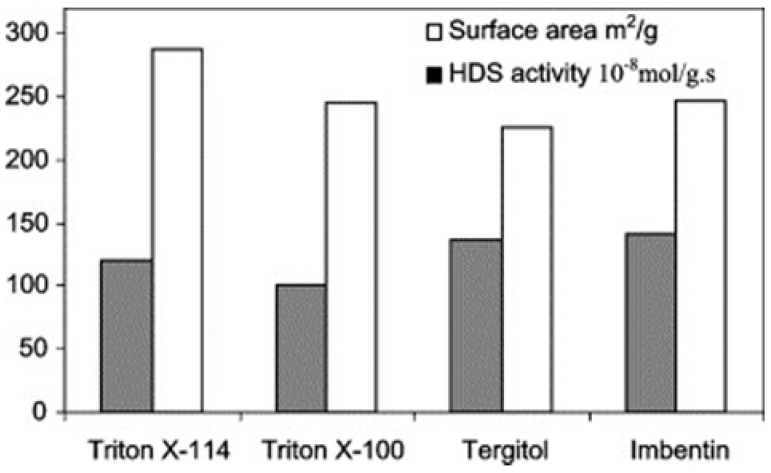
Thiophene HDS rate constants measured at 573 K and surface areas of the pre-catalysts prepared using different surfactants grades. Reproduced from reference [60] with permission from Elsevier.

The capping of sulfides with surfactants is an effective strategy to reduce the surface tension and thus prevent the possibility of aggregation and oxidation of nanoparticles. Highly dispersed Ni(Co)–Mo–S sulfides were prepared by simple room temperature solution reactions using nickel or cobalt salts and thiomolybdate precursors in the presence of nonionic surfactants [60]. Several substances of the same family of surfactants, different grades of Tergitol, Imbentin, or Triton, were tried as textural promoters. The length and the branching of the aliphatic tail, as well as the length of the polyethoxo fragment, were varied. Similar chemical compositions and very similar morphologies of the pre-catalysts were obtained. The variation in HDS activity as a function of the surfactant molecules used was minor (Figure 7), indicating that a wide variety of chemically similar surfactant molecules are suitable for such preparations [60].

The chemical stability of the formed nanoparticles will be improved and aggregation will be prevented with surfactants acting as stabilizing agents [16]. For instance, the cationic surfactant CTAB plays the role of a scaffold in the preparation of MoS_2_ monolayers with high surface areas [55]. The Ni-MoS_2_/Al_2_O_3_ composite coatings can be obtained by pulse electrodeposition [35]. The adsorption of surfactant CTAB on the particles has been proved to reduce the particle agglomeration, enhance the adhesion force to the cathode, enabling larger particles to be embedded in the composites. Therefore, the performance of microhardness and wear resistance of the Ni-MoS_2_/Al_2_O_3_ composite are improved. Trikalitis *et al*. synthesized mesostructured cobalt and nickel molybdenum sulfides with the surfactant alkyl-pyridinium (C*_n_*PyBr, *n* = 12, 14, 16, 18 and 20) acting as template [80]. The pore–pore separation represented by the *d*-spacing of the low angle Bragg peak of the sulfides is readily adjustable and increases with increasing surfactant chain length [80]. They also found that the surfactant decomposition would lead to the collapse of the mesostructured framework and the formation of an amorphous solid. Similar decomposition effect of the surfactants is also reported to change the particle size and aggregation state [17]. Further investigations are needed to clarify definitively the underlying mechanism of surfactant adsorption and characterize the arrangement of the surfactants at the surface of the sulfide nanoparticles, for the purpose to improve their catalytic and tribological properties.

### 3.2. Synthetic Fabrication of Molybdenum Sulfide with Polymers

Modification of inorganic nanoparticles with reactive polymeric ligands would incorporate chelating anchor groups, which allow the binding of functional molecules such as fluorescent dyes, as well as groups to tailor the solubility of the inorganic nanocrystals in solvents [18]. However, the role of polymers is diverse and detailed investigation are necessary [31]. In general, the various functions of polymers reported resemble those of small molcecular surfactants, such as template (nanoreactor), structure directer (kinetically controlled), and stabilizing agent (prevent aggregation or oxidation).

The polymers acting as templates or nanoreactors will provide novel morphologies and tunable sizes of the nanoparticles, which will be further modified with different functional groups, and easily removed after reactions. This subject has been well documented in recent review articles. Shi *et al*. reviewed the application of polyelectrolyte multilayer nanoreactors for generating diverse nanostructured materials [81]. Various parameters of the formed crystals can be easily tuned by changing the multilayer thickness, solution pHs, and polyelectrolyte layer composition. Recent advances in polymer-assisted fabrication of nanomaterials with emphasis on ordered polymeric nanostructures are reviewed by Liu *et al*. [19]. However, reproducibility of the synthesis was difficult to be achieved, since it was greatly influenced by the reaction time, nature of the reductant, acidity of the reaction solution, and reaction temperature. Even under identical experimental conditions, the size of the obtained samples was difficult to maintain identically.

The chemical stability of the sulfide-polymer composites would be enhanced. With the assistance of surface polymers, the sulfide nanoparticles gain improved stability against particle aggregation, since the Ostwald ripening is avoided. For a typical example, it was found that the DDP-MoS_2_ nanoparticles dispersed well in the organic solvent and were protected from oxidation in air because of the presence of the DDP layers [79]. Similarly, the dispersion capacity, oxidative stability, and antiwear ability of DDP-coated PbS and ZnS nanoparticles could be improved effectively by surface modification with DDP [82,83]. Zhang *et al*. suggested that the DDP capped MoS_2_ nanoparticles are much more stable than MoS_2_ particles in air [84], with improved lubrication property, load-carrying capacity, and solubility in organic solvents. While in another study, tribochemical changes are involved during the friction process of the DDP modified MoS_2_ LB film against steel [3], including the disordering and partial or even complete decomposition of the surface DDP, oxidation of MoS_2_ nanocores, with increased sliding cycles and temperatures.

Frequently, a habit of modification of inorganic crystals is ascribed to the selective adsorption of polymeric additives onto specified crystal faces, resembling the case of small molecular surfactants. Appropriate design of polymers as crystal modifiers would lead to a tailored architecture of inorganic materials [31]. PVP is used as a shape-control agent or crystal-habit modifier for the noble metals, promoting reduction onto specific crystal faces while preventing reduction onto others [16]. Cates *et al*. reported a biomimetic *in situ* synthesis of metal sulfides/polymer composites [85]. The surfactants used were suggested to mimic the soluble proteins found in biological systems, which have been shown to assemble at the surfaces of the polymer composite films, significantly affect nucleation and orientation of the growing crystals within the solid polymer composite films. The surfactants also cause aggregation of the inorganic particles into organized arrays within the composite films. The degree of control over crystallization of the inorganic phase critically depends on the strength of the chemical interactions between the surfactant headgroup, metal ions, and polymer matrix [85].

The fabrication with properly selected polymers would combine different functions of MoS_2_ particles and hydrocarbon chains. Polyaniline, poly(ethylene oxide) (PEO), poly(propylene glycol), poly-(vinylpyrrolidinone), methyl cellulose, polyethylenimine, polyethylene, and Nylon-6, were encapsulated into MoS_2_ [86]. The composites of (PEO)_0.92_MoS_2_ and (Polyaniline)_0.35_MoS_2_ show *p*-type metallic behavior, which will undergo transition from metal to insulator at low temperatures below 14 K and 9 K, respectively. The intercalation or encapsulation of polypyrrole between MoS_2_ layers *via* an *in situ* oxidative polymerization process has been realized [87]. Golodnitsky *et al*. developed a new type of composite thin film cathodes from the electroreduction of thiomolybdate anions [88]. They found that the addition of polymeric compounds, PEG and PEO of different molecular weights and concentrations, to the electrolytes significantly increase the nucleation process, improve the structural and electrochemical properties of the electrodeposited molybdenum sulfide cathode in three-dimensional Li-ion microbatteries.

The nanosulfides possessing organic modifiers are expected to have a wide range of applications in chemistry, bioscience and materials science. The key point to obtain highly active catalysts was the proper choice of the solvent and the organic admixture. The main advantage of these bioinspired routes is the combination of highly diverse organic chemistry and the highly flexible solution synthetic technique [16], enabling fabrication of sulfides with a broad range of desirable compositions, properties, and functionalities. The largest obstacles facing polymer-mediated preparation of nanocatalysts are compatible surface chemistry and shape retention. Ideally, fundamental catalytic studies should employ single crystalline surfaces that are clean and well characterized [16], while the polymer modified catalysts are protected by a layer of organic compounds. Although removing these agents may be required to create accessible active sites, it is difficult to do so without inducing significant morphological change *via* surface reconstruction, particle ripening, melting, or oxidation [16]. The structure characterization of the sulfide-organic interface is required with the purpose to understand the fabrication mechanism and thus optimize the catalytic, tribological, and mechanical performance of the nanocomposites [32]. Improvement of textural stabilities of the hybrid materials are needed, since their decomposition at high temperatures will result in collapsed of the structure. But more economic or greener strategy are needed, considering the large amounts of toxic organometallic compounds in the solutions, which make large scale preparations potentially expensive, time-consuming, and polluting.

## 4. Synthetic Fabrication of Molybdenum Sulfide with Inorganic Compounds

### 4.1. Synthetic Fabrication of Molybdenum Sulfide with Supports

The rational selection of support material is notably significant for the design of supported multifunctional nanoarchitectures of transition metal sulfides. Generally, the sulfides can easily be supported on any appropriate surface by co-impregnation, evaporation of its solutions, followed by calcination and sulfidation. Efforts have been made to achieve higher dispersion of the deposited active phase on the porous supports, since both theoretical and experimental studies have showed that supported sulfides are intrinsically more active than the randomly distributed sulfide particles [89].

It has been found that the surface orientation and crystallinity of supports, which differ from one support to another, would decide the microstructures of the sulfides. For hydrodesulphurization catalysts supported on alumina, silica-alumina, and titania, it is proposed that the difference in activity and synergistic effect is related to the MoS_2_ crystallite orientation on the surface of the support [90]. In a recent report [91], by means of atom-resolved scanning tunneling microscopy (STM) studies, it has been revealed that the equilibrium shape of single-layer MoS_2_ nanoparticles on a rutile TiO_2_ (110) support was decided by the strong particle-support interactions at the particle edges, and thus the specific orientation relative to the substrate was adopted. Sakashita *et al*. found that the differences in the surface oxygen arrangements among the (1 0 0), (1 1 0), and (1 1 1) crystal planes of γ-Al_2_O_3_ single crystal significantly affect the number and quality of Mo–O–Al linkages and the network of Mo atoms, resulting in different sulfidation temperatures, various dispersion and electrical states of MoS_2_ clusters [92].

There are also some cases where the catalytic properties of the material do not depend on the support morphology [93]. The incorporation of phosphate into the SBA-16 material led to decrease in coke formation in the final CoMoS/SBA-16catalysts, as opposite to the behavior of SBA-15-based counterparts [93].

It is well known that the support is usually not an inert carrier toward the active phase. The improvement of the HYD activity of the sulfide catalysts is due to a modified electronic effect of the Brönsted acidity of the support [10]. Bimetallic Co–Mo and Co–W sulfide HDS catalysts supported on SiO_2_ have been prepared by CVD technique, exposing presulfided MoS_2_/SiO_2_ and WS_2_/SiO_2_, respectively, to a vapor of Co(CO)_3_NO [94]. It was reported that the MoS_2_–O-support interactions will modify the electronic state, local structure, and intrinsic activity of Co–Mo–S on the edge. On the other hand, the interaction between the active phase or promoter ions and the support would be detrimental, leading to the lost of catalytic activity.

In some cases, the support itself will play the role of template. Hollow nanospheres of MoS_2_ and MoO_3_ have been prepared by sonochemical deposition on silica nanoparticles followed by hydrofluoric acid etching to remove the silica core [95]. With increased surface defects and accessibility, the hollow MoS_2_ spheres present superior HDS activity.

In an effort to obtain sulfides catalysts with better efficiency, one approach is to optimizing the interaction between the active sulfides and the supports surface [10,89,96]. However, characterization techniques almost reach their limits at this stage. As an effective and promising strategy, theoretical approaches are applied to model local structure and energy stability of the active phase-support interface and calculate the thiolysis and hydrolysis reaction energies for the metal–support linkages based on the density functional theory (DFT) combined with thermodynamic models and microkinetic models [97,98]. Recent advances accomplished in the area of theoretical simulation of catalysis by sulfides have been reviewed [97]. Costa *et al*. reported DFT investigation on the interaction of MoS_2_ layers on *γ*-alumina and anatase-TiO_2_ [89], comparing the thermodynamic stability of MoS_2_ and CoMoS anchored by S-edge and Mo-edge on these two supports under HDS conditions using Mo_5_CoS*_n_* clusters as model. Theoretical investigation shown that the calculated rank order of the supports with strong interaction had the tendency of SiO_2_ < carbon < Al_2_O_3_ < TiO_2_ < ZrO_2_ < Y_2_O_3_ [89,98], in agreement with the experimental results.

Recently, the influence of supports on the property of the sulfide active phase catalysts was reviewed in reference [99], where various supports, oxides (pure or mixed, basic or acidic), zeolites, carbon and clays were discussed. It was recognized that the use of titania and zirconia as supports could impart four to five times higher activities in Mo and W catalysts by comparison to alumina [10]. Both CoMoW/SBA-15 and CoMoW/SBA-16 catalysts were more active than a CoMo/Al_2_O_3_ commercial catalyst containing a small amount of phosphorous in its formulation [93]. The use of binary oxides [10,90], such as alumina-titania, silica–alumina, as supports would help in obtaining highly dispersed catalysts, avoiding disadvantages of certain supports, such as the low surface area of titania, small cavity sizes of zeolite.

Carbon supported catalysts, which show excellent catalytic activity, are favorable to lower coking deposition and easily recoverable from the waste catalysts by burning have attracted more and more attention. Hinnemann *et al*. found that MoS_2_ nanoparticles supported on graphite were a new class of electrode materials and promising catalyst for electrochemical hydrogen evolution at a moderate overpotential [100], using the criterion where the binding free energy of atomic hydrogen to the catalyst was close to zero. The nanoarchitecture of MoS_2_ overlayers supported on coaxial carbon nanotubes (CNT) has been successfully synthesized by a designed solution-phase route in the low temperature range [101]. The unique nanoarchitecture present high reversible capacity and excellent cyclability, which is one of the best cyclabilities so far reported for metal chalcogenides. The outstanding reversible lithium-storage behaviors is attributable to a synergy effect at the nanoscale between the CNTs core and the MoS_2_ sheath, and highlights the importance of the former in improving the lithium storage/release properties and maintaining thermodynamic/kinetic stability of the latter [101]. Future progress will focus on the design of excellent supports with high specific surface area, large pore size and pore volume. Optimization of the interactions between the support and the active phase are challenging to further improve the dispersion of sulfides for catalytic purpose.

### 4.2. Synthetic Fabrication of Molybdenum Sulfide with Promotion

Molybdenum sulfides are extensively used in refineries as heterogeneous hydrotreating catalysts to remove sulfur and nitrogen from oil fractions. Usually the promoted catalysts are obtained by the method of incipient wetness impregnation with salts of molybdenum and promoter. The nature of the promoter plays a determining role for the performance of the catalysts. The most efficient catalysts are always mixed binary, ternary, or even quaternary metal sulfides, among which ternary sulfides of cobalt and nickel are of considerable interest.

It is generally accepted that the most active catalyst is Co/Ni promoted MoS_2_ [8,26,35,41], with Co or Ni atoms located at the (h00) and (0k0) edges of MoS_2_ slabs (decoration model). The intrinsic activity of Co–Mo–S is correlated with the Co–S–Co antiferromagnetic interaction strength [94]. And the formation of dinuclear Co sulfide clusters on Mo(W)S_2_ edges regardless of the coverage of Co has been confirmed by the Co K-edge EXAFS analysis. As to the reaction mechanism, the HYD pathway is found to be catalyzed by sulfided species on the active phase, while the rate-determining step of the desulfurization pathway is catalyzed by vacant sites.

**Figure 8:**
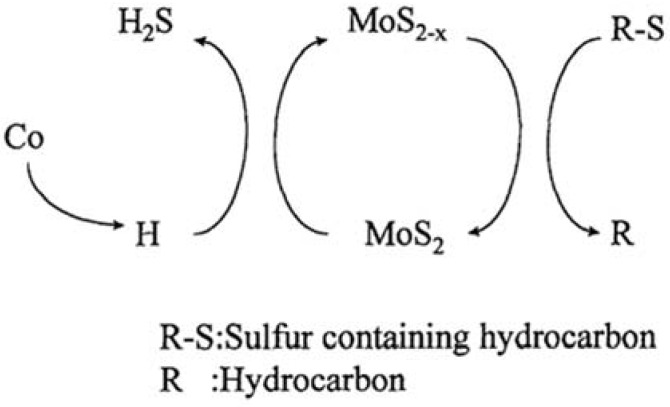
Scheme for the role of cobalt on HDS reaction**.** Reproduced from reference [102] with permission from Elsevier.

Progress has been made in understanding the basis for cobalt or nickel promoted MoS_2_ with improved hydrodesulfurization activity (HDS). Trikalitis *et al*. firstly reported the synthesis of templated mesostructured Co-Mo-S and Ni-Mo-S type materials by reacting MoS_4_^2^^－^ anions with Co^2+^ and Ni^2+^ in formamide solution in the presence of alkyl-pyridinium surfactant molecules [80]. The addition of cobalt to MoO_3_/Al_2_O_3_ accelerates the sulfiding of MoO_3_ and supply spillover hydrogen to sulfur atoms adsorbed on the HDS active sites and the resulting hydrogen accelerates the HDS reaction on the Mo sites (Figure 8) [102]. The sonochemically prepared catalysts of CoMoS/Al_2_O_3_, NiMoS/Al_2_O_3_, and CoNiMoS/Al_2_O_3_ are extremely active for the HDS of thiophene and dibenzothiophene, with activities several fold those of comparable commercial catalysts under identical conditions [50]. Grimblot reviewed the genesis, activation, architecture and nature of active sites of supported MoS_2_ hydroprocessing catalysts promoted by Co or Ni recently [103]. The role of the promoters was discussed. The different HDS catalytic activities for the promoted systems are explained by the distinct edge-wetting regimes inducing a significantly higher S-edge/Mo-edge ratio on the support, which is favorable for optimum promoter decoration [10].

Acid condensation of MoO_2_S_2_^2-^ anions yields nearly stoichiometric amorphous molybdenum oxysulfide MoOS_2_ [104], which is soluble in simple organics and possesses original microtubular morphology. This oxysulfide presents excellent HDS activity through an effective promotion with Co or Ni by using a non-ionic surfactant of the alkyl aryl-polyethylenglycol family (known as Tergitols or Tritons) as a scaffold. The reactions of MoS_4_^2^^－^and Mo_2_S_12_^2^^－^ anions with nickel or cobalt salts in mixed solutions produced amorphous Ni(Co)Mo solids with very high activity in the HDS of thiophene and 4,6-dimethyldibenzothiophene [60], where the Ni(Co)/Mo ratio depend on the conditions and particularly on the nature of the thiomolybdate anions applied. A series of Ni-Mo-B bimetallic amorphous catalysts have been prepared by chemical reduction of nickel nitrate and ammonium heptamolybdate with sodium borohydride aqueous solution [105]. There is a synergistic effect between Ni active site and Mo active site for phenol hydrodeoxygenation (HDO) reaction. The particle size became large with the decrease of Ni in the catalyst. The introduction of ultrasound will decrease the particle size, inhibit the formation of boron oxide, thus enhance the catalytic activity for phenol HDO.

The assistance of nitrilo triacetic acid (NTA) as chelating agent will enhance the decoration of the MoS_2_ slabs by the promoter atoms [106,107]. CoMo/alumina catalysts with constant Co and Mo contents have been prepared with the assistance of NTA [106]. Transmission electron microscopy analysis and infrared spectroscopy of adsorbed CO show that NTA has no effect on MoS_2_ dispersion, slab size and stacking, while the total amount of Co-promoted sites will be significantly increased. Comparison of activity and spectroscopic data implies that this increase accounts for the enhancement of HDS and HYD catalytic activity, and evidences the creation of different kinds of active sites. The NTA based NiMo catalyst supported on alumina has a fully promoted edge structure [107]. It is remarkable that NiMo catalysts could be fully restored to their initial state by resulfiding after thiophene HDS, whereas some active sites of the Mo catalysts will be irreversibly lost.

Potassium (K) promoted molybdenum sulfide supported on multi-wall carbon nanotubes (MWCNT) is reported recently to produce higher alcohols from synthesis gas [108]. It has been found that addition of K increases K–Mo–O interactions, decreases the Mo particle sizes, promotes the reducibility of the catalysts, increases the formation of alcohols and suppresses the formation of hydrocarbons.

Generally, the mixed sulfides are supported on certain porous substrates, and little attention is paid to the preparation of highly dispersed mixed sulfides. Future trends would be to obtain mixed sulfides in one simplified step *via* solution reaction using simple precursors, such as ammonium molybdate or molybdic acid, commercially available cobalt salts, cheap and nontoxic sulfur sources [26,60].

### 4.3. Synthetic Fabrication of Molybdenum Sulfide with Doping

Frequently, small amounts of impurities in the precursors have a profound influence on the morphology and property of the product. Many papers have been published on this subject. The introduction of more strongly active phases as dopant or as catalyst mixtures will increase the hydrogenating function of conventional industrial catalysts [10,109]. Ruthenium has been tested as a dopant for NiMo on alumina catalyst with an efficient promotion effect for the HDS of dibenzothiophene (DBT), and the HYD of tetralin [109]. The synergetic effects are ascribed to the formation of dual decoration sites of the MoS_2_ slabs, with the maximum activity obtained at a loading in the range of 0.25–0.5 wt % Ru.

Recently, sulfide catalysts stabilized in the hydrotreating environment contain structurally important carbon have attracted increasing attentions [57,110]. The carbon doped MoS_2_ can be obtained through thermal decomposition of the precursors containing some organic species. For example, coke containing MoS_2_ is obtained through decomposition of molybdenum naphthenate in the presence of either *n*-hexadecane or 1-methylnaphthalene as the hydrocarbon medium [110]. Moreover, it is supposed that the carbonaceous species dispersed well over the sulfide particles will prevent MoS_2_ slabs from sintering, affect the accessibility of reactants to MoS_2_ surface and consequently, influence the catalytic activity of the sulfides [60,110].

The role of the carbonaceous species on the activity of hydrotreating catalysts has been controversial for a long time. On one hand, the carbonaceous matter of any origin will prevent the crystallization of the sulfide [58]. In addition, carbon deposition is known to be one cause of catalyst deactivation, which is undesirable for further applications. But on the other, it appears that the textural and catalytic properties of the sulfides are greatly improved if small amounts of carbonaceous matter as binders and/or textural promoters are contained. The stabilizing role of carbon in doped sulfides has been investigated [57,60,111], which might be introduced in the form as well of oxygenated organic admixtures as of templating surfactants. The highly dispersed MoS_2_ or WS_2_ obtained by Afanasiev *et al*. with the presence of organic surfactant is stable up to 873 K [58]. They found that the sulfides contained 4−8 wt % of carbon impurity, which probably acts as a textural stabilizer to prevent the agglomeration of layered sulfide fringes. Such a positive effect of carbon for carbon-containing catalysts, with enhanced HDS catalytic activity (of at least 30%) and improved stability for gas–oil-treated samples, is also observed in the hydrotreatment of model compounds or gas–oil [111]. By using (NR_4_)_2_MoS_4_ (R = alkyl group) as a precursor, Berhault *et al*. reported the modifications of unsupported MoS_2_ *via* CH_3_-S-CH_3_ or dibenzothiophene treatment [57]. The increase of catalytic activity was related to the presence of alkyl groups in the precursor, which increased the inclusion of structural carbon in the operating catalysts. They suggested that the active transition metal sulfides were rather sulfide-supported transition metal carbides, namely sulfocarbide MoS_2-*x*_C*_x_*. The presence of carbon played an important role in increasing the dispersion of the active phase, and reducing both particle size and stacking number of the final Mo catalysts.

Recently, a new way of preparing active HDS catalysts is to add phosphate and a glycol to the impregnating solution [8]. Studies have shown that H_3_PO_4_ would react stoichiometrically with MoO_3_ to form diphosphopentamolybdate complexes. Glycol will interact with the basic OH groups and coordinatively unsaturated Al^3+^ sites on the phosphate-doped CoMo/Al_2_O_3_ [8], and therefore hinders the interaction between the cobalt-diphosphomolybdate species and the *γ*-Al_2_O_3_ support, leading to an efficient decoration of the MoS_2_ slabs by the promoter atoms. Briefly, there is a synergistic effect between phosphate and glycol, which have the beneficial effect to increase the solubility of the molybdenum salts, the stability of the impregnation solution, and finally, the catalytic performance of the supported catalysts.

Controversial results have been presented concerning the role of phosphorus doping for MoS_2_-based hydrotreating catalysts. For example, it was found that the HDS activity of Co-Mo-W supported on mesoporous SBA-15 and SBA-16 would be reduced by the presence of phosphorous [93]. Iwamoto *et al*. investigated the role of phosphorous on the textural, structural and catalytic properties of Mo based catalyst [112]. It was found that P and Mo atoms interacted strongly with equivalent sites of the alumina framework and the HDS activity was not promoted by P. In contrary, Nava *et al*. examined the effect of phosphorus addition on unsupported trimetallic Ni–Mo–W sulfide catalysts prepared by the *in situ* activation of nickel-containing tetramethylammonium thiomolybdotungstate [113]. They found that the presence of phosphorus had a negative impact on both textural and dibenzothiophene (DBT) HDS catalytic properties of the NiMoW catalysts compared with that of commercial NiMo/Al_2_O_3_ catalysts.

The impact of tin doping on the catalytic activity and selectivity of alumina-supported CoMoS was studied by Choi *et al*. [71], with the initial morphology of sulfide slabs remained unchanged. It has been shown that the olefin HYD activity decreased drastically with high tin loadings, while the thiophene HDS activity displayed a contrary tendency.

**Figure 9:**
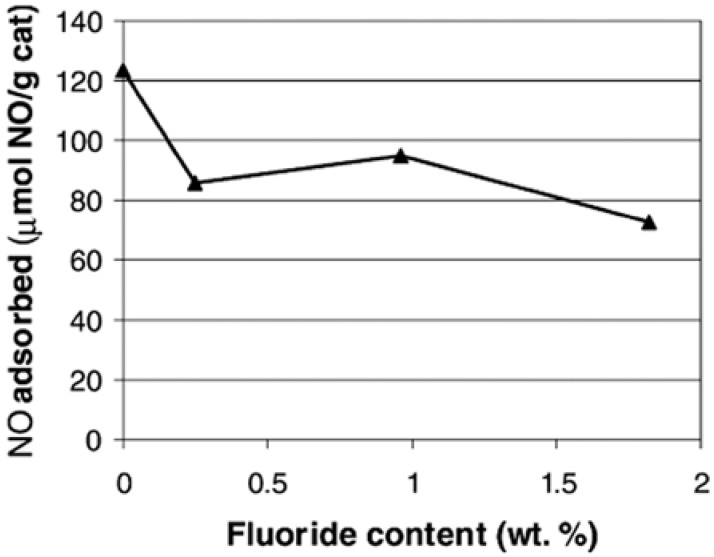
NO adsorbed as a function of fluoride content for sulfided Mo/Al_2_O_3_-F(*x*) catalysts. Reproduced from reference [114] with permission from Elsevier.

The incorporation of fluoride to the alumina support in Mo/Al_2_O_3_ catalysts will cause a significant drop in the isoelectric point of the alumina support [114], bringing about a negative effect on the electrostatic adsorption of Mo on the support during the impregnation step. The dynamic NO chemisorption experiments proved the drop in the dispersion of the MoS_2_ phase (Figure 9).

Trimetallic NMNiMo catalysts (NM = Pt, Pd, Ru) supported on alumina has been obtained through the incorporation of small amounts (1 wt %) of noble metals to NiMo/γ-Al_2_O_3_ catalysts [115]. Experiments have shown that the catalytic performance of the doped catalysts for 4,6-dimethyldibenzothiophene HDS depends on the nature of the noble metal. But there is no synergetic effect between noble metals and conventional NiMo/alumina catalysts, and the doping almost do not affect the textural characteristics, the dispersion and coordination state of the catalysts.

The introduction of doping elements is a promising strategy to realize a high level synthetic control, and reproducibly fine tuning of MoS_2_ properties by varying some synthesis parameters. It is also of fundamental interest to improve the preparation of a new class of industrial supports, as well as the use of properly selected additives to obtain a new generation of hydrotreating catalysts.

## 5. Synthetic Fabrication of Molybdenum Sulfide with Intercalation Chemistry

The intercalation chemistry of layered transition metal chalcogenides has been a subject of sustained interest. MoS_2_ has a sandwich interlayer structure formed by S-Mo-S layers, which are loosely bound to each other only by relatively weak van der Waals forces. Various applications of MoS_2_ arise from its highly anisotropic layered structure. The sulfide layers are negatively charged, so both electric charge and chemical species can be incorporated as guest species between the layers through electrostatic interactions [14,13,30,37,74,110,111]. The chemical and electrochemical intercalation chemistry of transition metal sulfides MX_2_ (M = Ti, Zr, Hf, Nb, Ta, Mo, W, V, and X = S) have been well investigated, because of their similar layer structure to those of graphite. This is an important feature of interest with regard to the intercalation and lubricant properties of MoS_2_ [72]. Strategies for successful intercalation are related to the improvement of both charge transfer and diffusion rates [14]. Furthermore, alkali-metal intercalation of inorganic fullerene-like structures showed respectable and time-invariant photoeffects, which could be applied for solar cells and the fabrication of inert scanning tunneling microscope (STM) tips [116].

**Figure 10:**
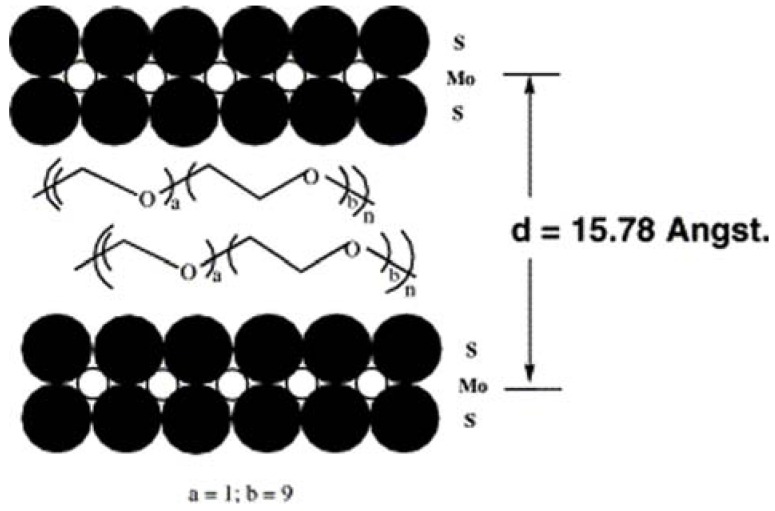
Schematic representation of the lamellar structure of (POMOE)_2.3_MoS_2_. Reproduced from reference [117] with permission from Elsevier.

Recently, the intercalation chemistry of MoS_2_ has been reviewed by Benavente *et al*. [14]. It is noteworthy that both MoS_2_ and WS_2_ show intercalation inertness toward most guest species, compared with metal disulfides of groups IV and V [48]. Generally, the intercalation could be realized only with powerful reducing agents (such as *n*-butyl lithium) or with the assistance of high energy irradiation (such as microwave). Therefore, most of the study is focus on the insertion of lithium and other alkali-metal ions [14,117,118,119]. The exfoliation of resulted lithiated MoS_2_ followed by reflocculation could be exploited to produce a variety of novel intercalation products [12,14,120,121], intercalated with simple inorganic (lithium, post-transition metals, metal hydroxides), organic (trichloroethylene, styrene, phenanthroline, alkylammonium cations, polymer, *etc.*), or organometallic (metallocenes, ruthenium hydroxoarene complexes, *etc.*) species. As an example, amine-MoS_2_ nanocomposites were prepared by addition of an aqueous solution of amine to the suspension of exfoliated MoS_2_, followed by stirring of the reaction mixture at 323 K for 48 h [122]. Poly[oxymethylene-(oxyethylene)] (POMOE) has been inserted in the lamellar structure of MoS_2_ by using the exfoliation and re-stacking property of LiMoS_2_ (Figure 10) [117]. The encapsulated polymer polyethylene oxide behaved as a solid electrolyte that could help in enhancing the diffusion of lithium from the anode to the cathode. The resulted intercalation compound (POMOE)_2.3_MoS_2_, an attractive material for lithium solid-state battery application, showed enhanced electronic conductivity due to a structural transformation of the MoS_2_ framework. The electrostatic interactions between the protonated guest species and the slightly negatively charged MoS_2_ sheets would give rise to the stability of the intercalated phase.

Studies about intercalated MoS_2_ with protonated 1,10-phenanthroline has been reported over the pH range of 0~11 [123]. A series of novel mesostructured lamellar MoS_2_ have been synthesized using a molten neutral *n*-alkylamine as the solvent as well as the template [48]. Such amine-intercalated phases can be transformed into mesoporous MoS_2_ by slow thermal treatments at 200 °C. Upon intercalation, the local coordination geometry of the Mo centers of the host lattice will change from trigonal prismatic to distorted octahedral (as in the metastable 1T-MoS_2_) [26]. Li *et al*. investigated the electrochemical property of MoS_2_ electrode and found that Mg^2+^ ions might be reversibly intercalated/deintercalated into the annealed MoS_2_ samples during the electrochemical charge/discharge processes [124], showing the possibility of MoS_2_ application in fabricating rechargeable magnesium batteries. Wang an coworkers have prepared two mesolamellar molybdenum sulfides, where the sulfur exists as S^2^^－^, S_2_^－^ and S_2_^2^^－^ species, with intercalated cetyltrimethylammonium surfactant cations at room temperature and under hydrothermal conditions, respectively [38]. Powder X-ray diffraction and transmission electron microscopy reveal that the materials have the interlayer distances of 34 and 28 Å, with the negatively charged sulfide layers being balanced by the intercalated cetyltrimethylammonium cations. But the mesolamellar compounds were thermally unstable, with the surfactant cations in the compounds start to decompose at about 200 °C [38], leading to amorphous sulfides.

The intercalated guest organic molecules are not necessarily electron donors. In some cases, the guest molecules behaving as pseudo-alkali metals could also be incorporated. Several examples have been published, such as a highly oriented, conducting film of restacked MoS_2_ containing ferrocene [125], MoS_2_ intercalated with substituted ferrocene [126], the intercalation complexes of dichalcogenides incorporating the metallocenes of cobalt and chromium [127].

Molecular species of greater complexity and size, such as metal clusters, may also be intercalated into MoS_2_. Bissessur *et al*. intercalated large molecular chalcogenide clusters of Co_6_Q_8_(PR_3_)_6_ (Q = S, Se, and Te and R = Alkyl) into MoS_2_ as well as other transition-metal dichalcogenides, such as TaS_2_, TiS_2_, NbS_2_, and MoSe_2_, by using their exfoliation and restacking property [13]. The intercalated phases had increased surface area, arising from the pores created by the pillaring effect of the cluster molecules. The synthesis of [Al_13_O_4_(OH)_24_(H_2_O)_12_]*_x_*MS_2_ (*x* = 0.02–0.05, M = Mo; *x* = 0.02–0.055, M = W) was accomplished by the intercalation of the aluminum cationic cluster [Al_13_O_4_(OH)_24_(H_2_O)_12_]^7+^ into sulfides [128]. According to the one-dimensional electron density mapping calculations and ^27^Al MAS-NMR analysis, the clusters maintained its structural integrity between the host layers while intercalated.

**Figure 11:**
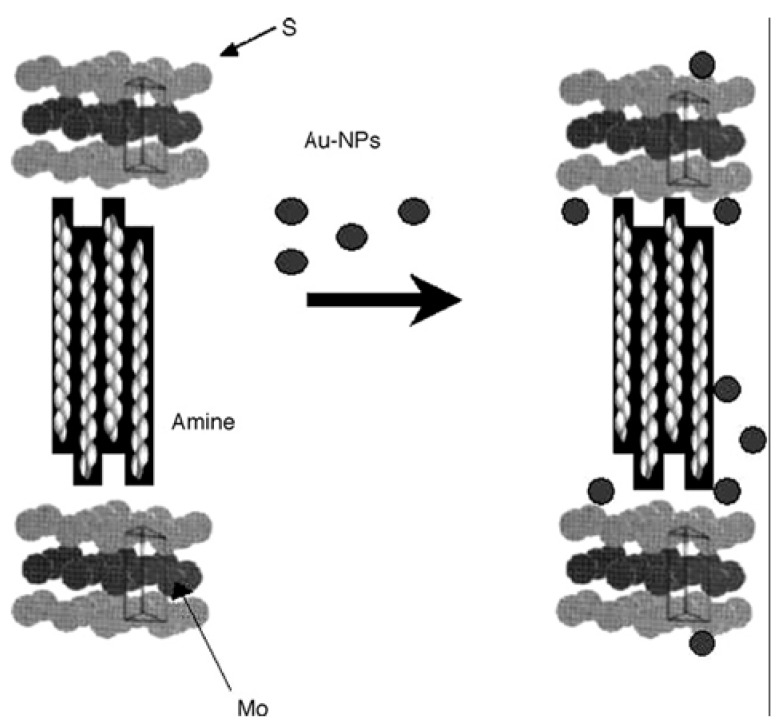
Schematic representation of the functionalization of the Li_0.1_MoS_2_(HDA)_2_ compound with gold nanoparticles and their position in the lamellar system. The MoS_2_ is represented in the 1T conformation. Reproduced from reference [122] with permission from Elsevier.

Lavayen *et al*. have successfully synthesized functionalized lamellar Li_0.1_MoS_2_(HDA)_2_ (HDA: hexadecylamine) nanocomposites with gold nanoparticles by chemical liquid deposition method (CLD). They proposed that the presence of oxygen groups (keto, hydroxo) of the surfactants would bring residual negative charge on the nanoparticles. The location of the gold nanoparticles was between the MoS_2_ lamella in the vicinity of the hydrophilic zones defined by the amine groups of the surfactants (Figure 11) [122].

Besides the intercalation of the desired species, the possibility of cointercalation of solvent molecules during the process should be considered simultaneously. Moreover, the intercalation of guest molecules will induce important changes in the structural, electronic, and thus the physico-chemical properties of the hosts. The nature of the products, the magnitude of the charge transfer, the structural and thermodynamic changes associated with the intercalation, as well as the reactivity of intercalates and the diffusion of the guest species in the interlaminar spaces are all important questions needs further investigation [14].

## 6. Concluding Remarks

This review summarizes the general strategies for synthetic fabrication of transition metal sulfide nanoparticles, with particular focus on those of MoS_2_. The above-cited works are just several examples and more detailed discussion is beyond the scope of this review. Numerous articles have been published on this subject, yet in most cases only some general characterizations are presented without much care about the chemical issues [26]. A general theory or a predictive empirical approach for the preparation and fabrication of transition metal sulfides is actually lacking. The role of the sulfide morphology in the hydrotreating activity of MoS_2_-based systems is still unresolved.

The challenge to develop catalysts with excellent hydrotreating properties based on MoS_2_ nanoparticles has been addressed mainly from three categories: fabrication with organic compounds, inorganic compounds, and intercalation chemistry. Although much work has been devoted to the subject, microstructure determination and control of the hybrid systems still remains a topical issue. Sometimes, the process of organic coating, inorganic doping, or guest intercalation takes place *in situ* with the preparation of the sulfide nanoparticles in one process or performs as a series of consecutive processes in one reactor. The complexity of the modified systems needs further elucidation of the mechanism during the process of synthesis, catalysis, *et al*., and thereby to improve the performance of nanocomposites both theoretically and experimentally. Currently, much of the property studies are about the tribological applications of MoS_2_. For the goal of industrial application, much more are needed to be considered, the costs of materials, stability of products, duration of equipments, and so on.

The preparation of nanostructured transition metal sulfides with defined composition and shape is of immense scientific and technological interest. Improvement and simplification of existing synthetic strategy are of significant importance to gain nanocrystal of sulfides with narrow size distribution, high crystallinity, and controllable surface properties or geometries. Such nanocrystals would exhibit unique properties, and subsequent utilization of them as building blocks for the fabrication of nanodevice is of significant interest.
